# Macronutrient intakes and associations with psoriasis severity: a cross-sectional analysis of the asking people with psoriasis about lifestyle and eating (APPLE) study

**DOI:** 10.1007/s00394-026-03914-y

**Published:** 2026-02-19

**Authors:** Sylvia Zanesco, Thiviyani Maruthappu, Christopher E. M. Griffiths, Ruotong Zhang, Kathryn V. Dalrymple, Rachel Gibson, Wendy L. Hall

**Affiliations:** 1 Department of Nutritional Sciences, King’s College London, London, UK; 2 Department of Dermatology, King’s College Hospital, King’s College London, London, UK

**Keywords:** Psoriasis, Skin, Diet, Nutrition, Macronutrients, Food groups

## Abstract

**Purpose:**

Nutrition in psoriasis management is an area of active research interest, but estimates of macronutrient intakes are lacking. The present study aimed to assess macronutrient intakes of people living with psoriasis in the UK and explore the relationship between their dietary sources and psoriasis severity.

**Methods:**

This was an online cross-sectional study collecting diet and psoriasis severity information from adults with psoriasis. Responses to a Food Frequency Questionnaire and the self-assessed Simplified Psoriasis Index were used to determine nutrient intakes and psoriasis severity.

**Results:**

Relative to Dietary Reference Values, participants with psoriasis (n = 257) reported an overconsumption of % energy from free sugars (median 11.2%, IQR 2.6–37.6) and an underconsumption of fibre (20.2 g/day, IQR 5.9–44.0). Compared to participants in the lowest quartile of intake, those in the highest quartile of intake for % free sugars from beverages were more likely to report high psoriasis severity (adjusted Odds Ratio (OR) 3.85, 95% CI 1.507–9.831, *P* trend = 0.04), although Body Mass Index (BMI) attenuated this relationship. When fully adjusted, including BMI, elevated intakes of % protein from total meat was associated with increased odds of reporting high psoriasis severity (OR 2.47, CI 0.984–6.196), whilst % protein intakes from plant-based sources (OR 0.36, 0.140–0.915) was inversely associated with reporting high disease severity; *P* trends ≤ 0.05.

**Conclusion:**

Prioritising plant-based foods may be beneficial to people living with psoriasis, but this hypothesis needs confirmation from randomised controlled trials.

**Supplementary Information:**

The online version contains supplementary material available at 10.1007/s00394-026-03914-y.

## Introduction

Psoriasis is a chronic inflammatory skin condition affecting 2% of the population in the United Kingdom (UK) [1]. People with psoriasis have an increased susceptibility to developing comorbid diseases such as obesity, type 2 diabetes (T2D), metabolic syndrome, and cardiovascular disease (CVD) [2], whose onset can potentially be influenced by dietary habits [3].

In the UK, unlike clinical guidelines for conditions such as CVD and T2D [4, 5], psoriasis management lacks dietary recommendations as an adjunct to medical treatment. However, dietary management is of importance to those living with the condition, where understanding the role of diet in managing psoriasis is the top research priority of the Psoriasis Priority Settings Partnership of the UK Psoriasis Association [6]. A recent Cochrane review highlighted the lack of high-quality, robust evidence from trials assessing the impact of diets on psoriasis symptoms [7]. In the absence of evidence-based dietary recommendations, psoriasis continues to be managed pharmacologically, with no authoritative nutrition advice, except for weight loss recommendations in adults with overweight or obesity [8].

There is limited knowledge regarding optimal macronutrient intakes in people with psoriasis, although several studies have characterised the nutritional intakes of those living with the condition. For instance, a retrospective analysis of the National Health and Nutrition Examination Survey (United States) revealed that individuals living with psoriasis (n = 156) reported significantly lower sugar intakes compared to those without psoriasis [9]. In contrast, a Japanese case–control study (n = 70) reported higher sugar consumption among individuals with psoriasis and psoriatic arthritis compared to matched healthy controls, with no significant differences observed in total fat, protein, or fibre intake [10]. Findings from an Italian case–control study of males with psoriasis (n = 41 cases) revealed higher intakes of total fat, total and simple carbohydrates, and lower intakes of fibre and protein compared to matched controls [11]. These findings are consistent with an Iranian case–control study (n = 45 cases), although this study included both males and females with psoriasis [12].

Prior to deriving nutrition recommendations, characterising diet and understanding areas of adequacy and improvement are paramount. To our knowledge, no study has examined the macronutrient intakes of people living with psoriasis in the UK, and we therefore aimed to:

- Describe macronutrient intakes of people with psoriasis for comparison with those of a nationally representative sample, and to compare with Dietary Reference Values (DRVs)
- Evaluate associations between sources of macronutrient intakes and disease severity

## Methods

### Study design

Diet, lifestyle, and psoriasis information were cross-sectionally collected from UK adult volunteers with psoriasis responding to an open web-based survey [13] as part of the Asking People with Psoriasis about Lifestyle and Eating (APPLE) study (NCT05448352) [14]. The APPLE study was approved by the King’s College London (KCL) Research Ethics Committee (REC) (LRS/DP-21/22-29257) and the London—Westminster National Health Service REC (23/LO/0536). This manuscript was written according to the Strengthening the Reporting of Observational Studies in Epidemiology—Nutrition (STROBE-NUT) [15] and the Checklist for Reporting Results of Internet E Surveys (STROBE-CHERRIES) [16] (Supplementary Tables 1–2). Appendix 1 describes the survey design, development, and metrics.

### Recruitment

Participants were recruited by convenience sampling between June 2022 and January 2024. The study was accessible on the study-specific landing page and was publicised on social media [17] and within the KCL fortnightly recruitment newsletter distributed to staff and students. No initial contact was made with potential participants. The UK Psoriasis Association (funding organisation) assisted with recruitment by inviting members of their research network, a member community interested in psoriasis research, to participate in the study and by advertising the study on their social media platforms.

### Psoriasis severity

Participants self-reported their psoriasis severity using the self-assessed Simplified Psoriasis Index (sa-SPI) integrated into the survey. The sa-SPI is a validated scoring measure generating a psoriasis severity score between 0 and 70 points based on severity, psychosocial impact, and intervention history components [18]. Scores between 0 and 9 points were considered mild psoriasis, 10 – 19 points as moderate psoriasis, and 20 – 70 points as severe psoriasis.

### Demographic and lifestyle variables

Age, sex, weight, height, and smoking status were self-reported. Weight and height were used to calculate Body Mass Index (BMI). Participants were considered to have a psychological morbidity if “Yes” was selected for “Depression” or “Anxiety” concerning the question “Have you ever been medically diagnosed with any of the following conditions?”. The Alcohol Use Disorders Identification Test Consumption (AUDIT-C) evaluated alcohol use by scoring the frequency, units, and over-consumption of alcohol using a 5-point Likert scale for a maximum of 12 points [19].

### Dietary assessment

Total energy intakes (kcal/day) were determined from participant responses to a modified European Prospective Investigation into Cancer and Nutrition (EPIC) Food Frequency Questionnaire (FFQ), considering intakes over the past 12 months. Intakes of < 500 or > 3500 kcal/day for females and < 800 or > 4200 kcal/day for males were omitted from the analysis [20]. Modifications to the original 131-item EPIC FFQ [21] included omitting 4 food items and introducing 20 items for a total of 147 items [22]. Frequency responses were converted to portion fractions and were multiplied by standard portion sizes to calculate the weight of food per day (g/day) for each food item. The energy content (kilocalories) and macronutrient composition (protein, total metabolisable carbohydrate (excluding fibre), total sugars, free sugars, total fat and fractions of dietary fat, and fibre (Association of Official Analytical Chemists (AOAC)) of each food item was obtained using the Composition of Food Integrated Dataset (CoFID) [23] and where data was missing the FoodCentral database was used instead [24]. Free sugars were calculated for each food item using the Scientific Advisory Committee on Nutrition (SACN) definition of “monosaccharides and disaccharides added to foods by the manufacturer, cook or consumer, plus sugars naturally present in honey, syrups, and unsweetened fruit juices. Under this definition, lactose, when naturally present in milk and milk products, is excluded” [25]. If unavailable, the *cis*-monounsaturated fatty acids (MUFAs) per 100g of food were determined by subtracting *trans*-MUFAs from the total MUFA content per 100g of the given food.

### Quantifying macronutrient intakes

Intakes of protein, carbohydrates, starch, total sugars, free sugars, total fat, saturated fat (SFA), total MUFAs, *cis-* and *trans*-MUFAs, total polyunsaturated fatty acids (PUFAs), *n*-6 and *n*-3 PUFAs, were expressed in contribution to energy as percentages of total energy intake. AOAC fibre was reported as an absolute value in g/day and converted into g/1000 kcal for statistical analyses, where the total daily fibre intake (g/day) is multiplied by 1000 kcal and divided by the total energy intake kcal/day.

### Comparisons with UK government guidelines

Macronutrient intakes of the study population were compared with UK government recommendations. For carbohydrates and fats, UK guidelines are reported as DRVs, expressed as a percentage of total energy intake per day. Percentage of total energy intakes of total fat, SFA, MUFAs, *n*-3 and *n*-6 PUFAs, free sugars and g/day of fibre were dichotomised into intakes above or below the adult DRVs and expressed as the frequency (n) and proportion (%) of participants meeting, or not meeting, the set DRVs [25, 26].

### Comparisons with the UK population

Macronutrients reported as median (95% Confidence Intervals (CI)) per day, were compared with the intakes (from food sources only) of the general UK population using data from the National Diet and Nutrition Survey (NDNS); a rolling programme (RP) and cross-sectional study in the UK that collects representative information on the nutritional intakes of the UK population [27]. The diet assessment methodology of the NDNS is based on a series of 4-day food diaries. Intakes of the APPLE study population were compared to the NDNS RP Adult participants (between 19 and 64 years old) from Years 9–11.

### Macronutrient sources

Since the quality and source of dietary macronutrients may determine their relationships with health outcomes, macronutrient intakes found to be significantly correlated with psoriasis severity were further categorised into the proportions derived from food groups (Appendix 2). The sources of macronutrient intakes were expressed as a percentage contribution of the food group towards the total intake of that macronutrient. For example, % protein from red meat was calculated as the sum of protein from red meat sources (g/day) / total protein (g/day) × 100. Protein intakes from total meat, red meat, processed meat, poultry, fish, high-fat dairy, low-fat dairy, legumes, nuts, and eggs were calculated [28]. Fibre intakes were calculated from fruits, vegetables, potato products, wholegrain cereals, and non-wholegrain cereals as defined in a previous UK population study [29], with the addition of fibre from tree nuts. Free sugars were calculated from desserts and puddings, beverages, added sugars and condiments, and breakfast cereal. Intakes of *n*-6 PUFAs were calculated from nuts, fat-based spreads, red meat, processed meat, poultry, and eggs.

#### Statistical analysis

Data was visually examined for normality using Q-Q plots and histograms. Information on demographic, anthropometric, and lifestyle characteristics was tabulated into descriptive statistics reporting the median (interquartile range [IQR]) for continuous variables, and frequency (n) and proportion (%) for categorical variables. The consumption (in g/day) of food groups was reported per tertile of psoriasis severity as mean (standard deviation [SD]). Macronutrient intakes were dichotomised to report the frequency (n) and proportion (%) of participants whose dietary intakes align with the adult DRVs.

Macronutrients were reported as the median (95% CI) to compare with NDNS data. Variables from the NDNS Year 9–11 RP were corrected using weights to adjust for socioeconomic status and non-responsiveness to reduce selection and non-response bias [30]. The weight factor used was wti_Y911 (Weight for individual and diary-all ages, combined Y9-11) to analyse macronutrient intakes of the NDNS population. Supplementary Table 3 provides the demographic characteristics of the representative UK population sample from the NDNS (n = 1392).

To obtain parametric distributions, non-parametric variables were fractionally ranked and transformed using the Inverse Distribution Function (IDF). Correlations between food groups and macronutrients with psoriasis severity were examined with Pearson’s correlation coefficient. Associations between macronutrient source and psoriasis severity were reported as the Odds Ratio (OR) and 95% CI, obtained from multinomial logistic regressions with *P* for trend analyses. Macronutrient intakes (by food group source) were evaluated in quartiles, with the lowest intake category (Q_1_) as the reference, and Q_4_ as the highest category. Models were adjusted in an additive sequence of covariates. Model I included age (years, continuous), sex (male or female), and smoking status (active smoker or non-smoker). Model II included model I plus the AUDIT-C score (continuous). Model III included model II plus psychological morbidity (yes or no). Model IV included model III and BMI (continuous). *P* < 0.05 was considered statistically significant. Analyses were performed with IBM SPSS Statistics (version 29.0.0.0) and R software (version 4.43) (for the trend analyses).

## Results

### Survey responses

**Fig. 1:**
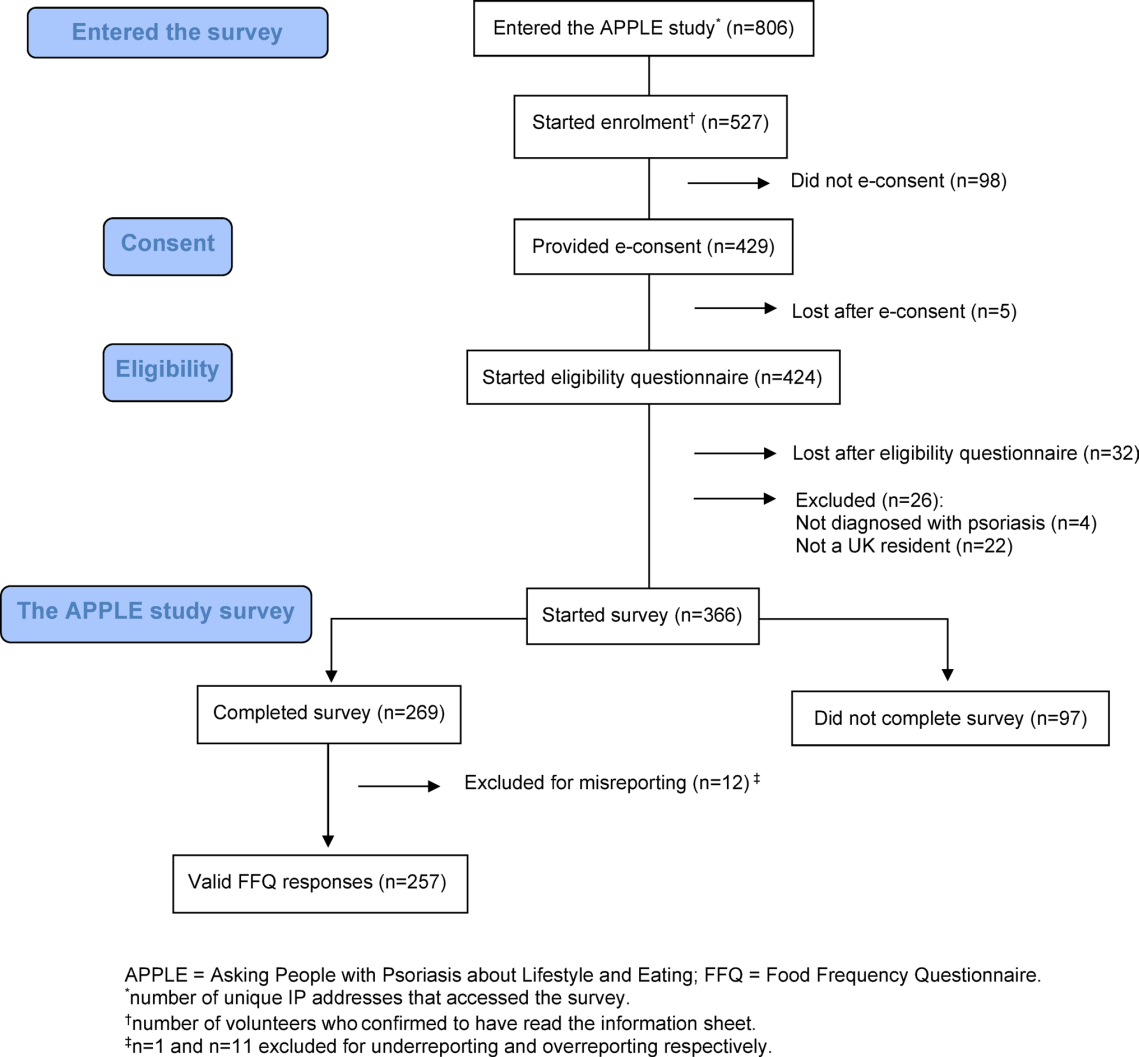
CONSORT flow chart

Figure 1 illustrates the CONSORT flow diagram for the APPLE study. There were 806 unique site visitors, of which 429 provided consent, and 366 participants started the APPLE study survey. This translates into a view rate (the number who started enrolment out of unique site visitors) of 65% and a participation rate (the number who e-consented out of those who started enrolment) of 81%. Ninety-seven participant responses (27%) were excluded as their survey response was terminated partway through and therefore did not complete the FFQ. A further twelve entries (4%) were excluded from the nutrition analyses for misreporting energy intakes [20]. Data was analysed from the remaining 257 participants with complete survey responses, for a completion rate of 60% (number completed out of total number consented).

### Demographic characteristics

The demographic characteristics of participants with valid survey responses are presented in Table 1. Participants were predominantly non-smoking (82%), females (83%), of white-British ethnicity (85%), with a median (IQR) psoriasis severity score of 15 (12). The median (IQR) age was 40 (20) years, and BMI was 25 (8) kg/m^2^, with 51% categorised as having overweight or obesity. Approximately two-thirds (60%) of participants were at a low risk of alcohol over-consumption. The most prevalent comorbidities were anxiety (41%), depression (25%), and psoriatic arthritis (21%) (Supplementary Table 4).

**Table 1:** The demographic characteristics of APPLE study participants with valid survey responses (n = 257)

Age, years (median, IQR)	40 (20)
Sex (n, %)	
Male	45 (17.5)
Female	212 (82.5)
BMI (median, IQR)	25 (8)
Body Mass Index (BMI) classification (n, %)	
Underweight	7 (2.8)
Healthy weight	118 (46.5)
Overweight	67 (26.4)
Obesity	62 (24.3)
Ethnicity (n, %)	
White-British	218 (84.8)
White (Other)	16 (6.3)
Mixed	10 (3.8)
South Asian	9 (3.5)
Asian (Other)	3 (1.2)
East Asian	1 (0.4)
Smoking status (n, %)	
Non-smoking	211 (82.1)
Actively smoking	45 (17.5)
Preferred not to say	1 (0.4)
Alcohol overconsumption (n, %)	
Low risk of dependency	154 (59.9)
Increasing risk of dependency	80 (31.1)
Higher risk of dependency	20 (7.8)
Possible dependence	3 (1.2)
Family history of psoriasis (n, %)	
Yes	137 (53.3)
No	120 (46.7)
Psoriasis severity (median, IQR)	15 (12)
Psoriasis severity (n, %)	
Mild	62 (24.1)
Moderate	115 (44.7)
Severe	80 (31.2)

n = 3 missing values for BMI due to lack of completeness

Underweight BMI < 18.50 kg/m^2^; normal weight BMI ≥ 18.50 kg/m^2^ and ≤ 24.99 kg/m^2^; overweight BMI ≥ 25.00 kg/m^2^ and ≤ 29.99 kg/m^2^; obesity BMI ≥ 30.00 kg/m^2^

Risk of alcohol overconsumption was determined using the Alcohol Use Disorders Identification Test Consumption: low risk of dependency, 0–4 points; increasing risk of dependency, 5–7 points; higher risk of dependency, 8–10 points; possible dependence, 11–12 points

Psoriasis severity was evaluated using the self-assessed Simplified Psoriasis Index; mild psoriasis, 0.00–9.99 points; moderate psoriasis, 10.00–19.99 points; severe psoriasis, > 20.00 points

### Comparisons of macronutrient intakes with dietary reference values and with the UK population per the NDNS

As shown in Table 2, median intakes (% energy) for total fat, SFA, and total, *cis* and *trans*-MUFAs were similar in both the APPLE study and UK populations. Approximately two-thirds of the APPLE study population (70%) and the general UK population (64%) exceed the upper recommended intake for SFA (% energy). Intakes of fatty acid subtypes generally aligned with DRVs in both populations, including *trans*-MUFAs (100% below the upper recommended intake as % energy in both populations), total *n*-3 PUFAs (96% vs 100% met the DRV), and total *n*-6 PUFAs (99% vs 100% met the DRV). Most of the APPLE study population (78%) and a greater proportion of the UK population (93%) do not meet the DRV for daily fibre intake, with median intakes across both population groups approximating 19–20 g/day. Median percentage energy intakes of free sugars are twice the UK recommendations (≤ 5% energy), with the APPLE study population reporting 11% of total energy and 10% reported for the UK population. Only 12% and 15% of the APPLE study population and the UK population, respectively, were within the UK upper recommended limit for free sugar intakes. Supplementary Table 5 compares food group intakes between the NDNS and the APPLE study populations.

**Table 2:** Median macronutrient intakes of study participants and proportions of participants that met Dietary Reference Values, with comparison to a nationally representative population of adults aged 19–64 years from the National Diet and Nutrition Survey, Years 9–11

Macronutrient	DRV	^*^APPLE (n = 257)			^†^ NDNS (n = 1392)		
		DRV met n (%)	DRV not met n (%)	Median (2.5th, 97.5th percentile)	DRV met n (%)	DRV not met n (%)	Median (2.5th, 97.5th percentile)
Energy, kcal/day	–	–	–	1666.2 (743.8, 3035.7)	–	–	1764.0 (876.6, 3061.5)
Protein, % energy	–	–	–	15.8 (10.2, 24.9)	–	–	16.3 (10.6, 26.7)
Total fat, % energy	≤ 35%	136 (52.9)	121 (47.1)	34.5 (22.1, 51.2)	776 (55.7)	616 (44.3)	34.1 (20.3, 46.6)
Saturated fat, % energy	≤ 10%	77 (30.0)	180 (70.0)	11.8 (6.0, 20.3)	500 (35.9)	892 (64.1)	12.6 (6.0, 19.9)
Total MUFAs, % energy	≥ 13%	160 (62.3)	97 (37.7)	13.9 (8.5, 21.6)	598 (42.9)	794 (57.1)	^‡^12.6 (7.2, 18.4)
*trans*-MUFAs, % energy	≤ 2%	257 (100.0)	0 (0.0)	0.41 (0.13, 1.06)	1392 (100.0)	0 (0.0)	0.45 (0.12, 0.96)
Total *n*-3 PUFAs, % energy	≥ 0.2%	244 (96.1)	13 (3.9)	0.50 (0.17, 1.40)	1392 (100.0)	0 (0.0)	^§^0.94 (0.43, 2.34)
Total *n*-6 PUFAs, % energy	≥ 1%	254 (98.8)	3 (1.2)	2.64 (1.11, 8.97)	1392 (100.0)	0 (0.0)	^‖^4.74 (2.47, 8.91)
Fibre, g	≥ 30	56 (21.8)	201 (78.2)	^¶^20.2 (5.9, 44.0)	102 (7.3)	1290 (92.7)	^¶^18.5 (7.3, 39.8)
Total carbohydrate, % energy	–	–	–	49.0 (29.9, 68.8)	–	–	48.4 (31.4, 64.6)
Free sugars, % energy	≤ 5%	30 (11.7)	227 (88.3)	11.2 (2.6, 37.6)	214 (15.4)	1178 (84.6)	9.7 (2.2, 24.6)

Median (2.5th and 97.5th percentiles) expressed as a % of total energy

DRV = Dietary Reference Value; APPLE = Asking People with Psoriasis about Lifestyle and Eating; NDNS = National Diet and Nutrition Survey; MUFAs = Monounsaturated Fatty Acids; PUFAs = Polyunsaturated Fatty Acids

^*^ Intakes estimated using a FFQ; † Intakes estimated using 4-day food diaries; ‡ *cis*-MUFAs; § *cis n-*3 PUFAs; ‖ *cis n*-6 PUFAs; ¶ Fibre reported as Association of Official Analytical Chemists (AOAC) fibre

### Macronutrient intakes by psoriasis severity

The mean (SD) macronutrient intakes (as % energy) by tertiles of psoriasis severity with Pearson’s correlation coefficients are tabulated in Table 3. Free sugars were positively correlated with psoriasis severity (*r* = 0.161, *P* = 0.010). Fibre (*r* = − 0.183, *P* = 0.003)*, n*-6 PUFAs (*r* = − 0.152, *P* = 0.015), and total PUFAs (*r* = − 0.132, *P* = 0.036) were negatively correlated with psoriasis severity. There were no further correlations with the other macronutrients assessed.

**Table 3:** Mean (SD) macronutrient intakes (as % energy) and Pearson's correlation coefficients with psoriasis severity (self-assessed Simplified Psoriasis Index)

Macronutrient (% energy)	Psoriasis severity				Correlations	
	T_1_ 6.3 (4.8)	T_2_ 16.7 (2.4)	T_3_ 27.2 (5.0)	All	*r*	*P*
Protein	15.8 (3.3)	16.4 (3.6)	16.6 (4.4)	16.0 (3.5)	0.113	0.071
Total carbohydrate	49.7 (10.0)	49.2 (8.6)	49.1 (10.5)	50.3 (9.0)	-0.048	0.443
Total sugars	23.5 (8.1)	23.8 (7.8)	23.7 (8.6)	24.0 (8.3)	0.015	0.812
Free sugars	11.5 (8.6)	13.9 (8.0)	14.2 (8.5)	13.9 (8.3)	0.161	**0.010**
Total fat	35.0 (7.5)	35.5 (6.5)	34.5 (7.7)	34.5 (6.9)	0.008	0.894
Saturated fatty acids	11.7 (3.4)	12.0 (3.5)	12.1 (3.4)	12.0 (3.5)	0.092	0.144
Total MUFAs	14.1 (3.4)	14.5 (2.8)	13.7 (3.4)	13.9 (3.1)	0.008	0.895
*cis*- MUFAs	13.6 (3.4)	14.0 (2.8)	13.2 (3.3)	13.4 (3.0)	0.006	0.925
*trans*-MUFAs	0.4 (0.2)	0.4 (0.2)	0.5 (0.2)	0.4 (0.2)	0.155	**0.013**
Total PUFAs	6.2 (2.1)	6.0 (2.1)	5.6 (2.1)	5.8 (2.0)	− 0.132	**0.036**
*n*-6 PUFAs	3.4 (1.8)	3.3 (1.7)	2.8 (1.7)	3.0 (1.7)	− 0.152	**0.015**
*n*-3 PUFAs	0.6 (0.2)	0.6 (0.3)	0.6 (0.2)	0.6 (0.2)	0.040	0.547
Fibre^*^	23.6 (10.5)	22.9 (10.6)	20.3 (10.4)	23.0 (10.5)	− 0.183	**0.003**

The mean (standard deviation) of the macronutrients is expressed as a % of the energy intake (transformed using the Inverse Distribution Function) by tertiles of psoriasis severity. *P *< 0.05 was considered statistically significant.

Psoriasis severity was determined using the self-assessed Simplified Psoriasis Index

MUFAs = Monounsaturated Fatty Acids; PUFAs = Polyunsaturated Fatty Acids; *n* = omega

^*^Association of Official Analytical Chemists (AOAC) fibre reported in g/day

### Food group sources of macronutrients by psoriasis severity

Supplementary Table 6 presents the mean (SD) contribution of macronutrients from food groups as a percentage of the total intake for a given macronutrient, with Pearson correlation coefficients for the relationship between percentage macronutrient intake (from food groups) and psoriasis severity. Positive correlations with psoriasis severity emerged for % protein from total, processed, red, and poultry meats, % *n*-6 PUFAs from processed meat and poultry, % fibre from potato products and non-wholegrain cereals, and % free sugars from beverages (*r* between 0.122 and 0.228, *P* < 0.05). Negative correlations were revealed for % protein from plant-based sources and nuts, % fibre from legumes and nuts, % *n*-6 PUFAs from nuts, and % free sugars from desserts and puddings (*r* between − 0.222 and − 0.123, *P* < 0.05).

Table 4 reports the OR (95% CIs) of reporting a *high* psoriasis severity (T_3_ of sa-SPI score) across quartiles of % macronutrient intakes from food groups adjusted for covariate models III and IV. Unadjusted and adjusted OR (95% CIs) for models I-II are tabulated in Supplementary Table 7. Supplementary Table 8 shows the unadjusted and adjusted OR (95% CIs) of reporting an *increasing* psoriasis severity (T_2_ of sa-SPI score) across quartiles of % macronutrient intakes from food groups for unadjusted and adjusted models (I-IV).

**Table 4:** Adjusted Odds Ratios (95% Confidence Intervals) of reporting *high* severity (T_3_, n = 88) compared to low psoriasis (T_1_, n = 83) severity by quartiles (Q_1_-Q_4_) of protein, fibre, and free sugar intakes from food groups (models III-IV)

	Mean (SD) and upper and lower limits of the percentage weight of macronutrient intakes derived from food groups (quartiles)				
	Q_1_	Q_2_	Q_3_	Q_4_	*P* trend
% protein from total meat	4 (9) 0–15	21 (4) 15–27	33 (4) 27–39	50 (9) 39–76	
Number with high psoriasis severity out of the total number per quartile of intake	18/64	14/63	28/64	28/64	
Model III	Ref	0.89 (0.361 – 2.196)	3.20 (1.310 – 7.803)	2.94 (1.216 – 7.116)	**0.002**
Model IV	Ref	0.75 (0.298 – 1.888)	2.47 (0.984 – 6.196)	2.01 (0.785 – 5.136)	**0.028**
% protein from red meat	0 (3) 0–3	6 (2) 3–10	13 (2) 10–16	22 (5) 16–37	
Number with high psoriasis severity out of the total number per quartile of intake	20/65	14/63	26/63	28/64	
Model III	Ref	0.65 (0.266 – 1.574)	2.11 (0.871 – 5.088)	2.56 (1.051 – 6.234)	**0.006**
Model IV	Ref	0.55 (0.223 – 1.374)	1.56 (0.623 – 3.909)	1.81 (0.711 – 4.625)	0.069
% protein from PBS	0 (2) 0–2	5 (2) 2–8	11 (2) 8–15	20 (4) 15–34	
Number with high psoriasis severity out of the total number per quartile of intake	29/64	22/64	19/63	17/64	
Model III	Ref	0.45 (0.176 – 1.163)	0.32 (0.129 – 0.813)	0.29 (0.112 – 0.727)	**0.007**
Model IV	Ref	0.47 (0.181 – 1.245)	0.36 (0.140 – 0.915)	0.39 (0.147 – 1.026)	**0.050**
% protein from nuts	0 (1) 0–1	3 (1) 1–4	6 (1) 4–8	11 (3) 8–19	
Number with high psoriasis severity out of the total number per quartile of intake	28/63	23/64	25/64	12/64	
Model III	Ref	0.51 (0.207 – 1.250)	0.84 (0.332 – 2.097)	0.18 (0.072 – 0.478)	**0.002**
Model IV	Ref	0.51 (0.205 – 1.287)	0.98 (0.380 – 2.505)	0.24 (0.092 – 0.651)	**0.019**
% fibre from potatoes	0 (4) 0–5	8 (2) 5–10	13 (2) 10–16	21 (4) 16–33	
Number with high psoriasis severity out of the total number per quartile of intake	14/64	22/64	30/64	22/63	
Model III	Ref	1.20 (0.495 – 2.926)	3.13 (1.243 – 7.902)	1.91 (0.742 – 4.916)	0.058*
Model IV	Ref	1.12 (0.455 – 2.769)	2.53 (0.979 – 6.524)	1.43 (0.535 – 3.803)	0.257
% fibre from NWG cereals	1 (3) 0–4	7 (1) 4–9	11 (1) 9–13	17 (3) 13–26	
Number with high psoriasis severity out of the total number per quartile of intake	17/64	24/62	21/65	26/64	
Model III	Ref	1.52 (0.642 – 3.616)	2.29 (0.908 – 5.751)	2.83 (1.089 – 7.333)	**0.021**
Model IV	Ref	1.36 (0.564 – 3.300)	1.81 (0.703 – 4.664)	2.28 (0.856 – 6.058)	0.082
% fibre from nuts	0 (1) 0–1	2 (1) 1–4	5 (1) 4–7	10 (2) 7–16	
Number with high psoriasis severity out of the total number per quartile of intake	26/62	23/65	25/65	14/63	
Model III	Ref	0.53 (0.218 – 1.311)	0.89 (0.368 – 2.153)	0.31 (0.123 – 0.780)	**0.043**
Model IV	Ref	0.58 (0.232 – 1.462)	0.93 (0.378 – 2.311)	0.37 (0.143 – 0.942)	0.095
% free sugars from beverages	0 (12) 0–15	24 (5) 15–32	41 (5) 32–49	64 (12) 49–100	
Number with high psoriasis severity out of the total number per quartile of intake	14/64	28/64	16/64	30/63	
Model III	Ref	3.70 (1.495 – 9.171)	1.42 (0.560 – 3.594)	3.85 (1.507 – 9.831)	**0.040**
Model IV	Ref	2.98 (1.178 – 7.544)	1.04 (0.397 – 2.724)	2.96 (1.127 – 7.780)	0.163

PBS = Plant-based sources; NWG = non-wholegrain

Q_1_ = lowest intake [referent quartile (Ref.)]. Q_4_ = highest intake

Percentage macronutrient intake from food groups is expressed as the mean (SD) for each quartile of intake calculated as a proportion of the total intake for that specific macronutrient, e.g. % protein from red meat was calculated as the sum of protein from red meat sources (g/day) / total protein (g/day) × 100

Multinomial logistic regression determined the Odds Ratios (95% Confidence Intervals) of reporting *high* psoriasis severity with *P* for trend to detect significant linear relationships with intakes. *P* < 0.05 was considered statistically significant.

Confounder adjustment models:

Model I = age (continuous), sex (male or female), and smoking (yes or no)

Model II = Model I + Alcohol Use Disorders Identification Test Consumption score (continuous)

Model III = Model II + psychological morbidity (yes or no)

Model IV = Model III + Body Mass Index (continuous)

^*^ % fibre from potatoes was statistically significant until adjustment for Model III, which included psychological morbidity

### Protein

Compared to participants in the lowest quartile of intake (Q_1_) for % protein from red meat, those in the highest quartile (Q_4,_ mean 22% of total protein intake from red meat) were more likely to report *high* psoriasis severity (OR = 2.56, 95% CI 1.051 – 6.234, *P* for trend = 0.006), although adjustment for BMI attenuated this association. After adjusting for all potential confounders inclusive of BMI (Model IV), participants in Q_3_ for % protein from total meat (mean 33% of total protein intake from meat sources), were twice as likely to report *high* psoriasis severity (OR = 2.47, 95% CI 0.984 – 6.196, *P* for trend = 0.028), whilst those in Q_3_ for % protein from plant-based sources (mean 11% of total protein intake from plant sources) were less likely to report *high* psoriasis severity (OR = 0.36, 95% CI 0.140 – 0.915). Participants in the highest quartile (Q_4_) for % protein from nuts (mean 11% of total protein intake from nut sources) were associated with a lower likelihood of reporting *high* psoriasis severity (OR = 0.24, 95% CI 0.092 – 0.651), all *P* values for trend were ≤ 0.05.

### Fibre

When comparing the highest (Q_4_) versus the lowest quartiles (Q_1_) of % fibre intakes, an increased likelihood of reporting a *high* psoriasis severity was revealed in those who obtained a greater proportion of their dietary fibre from refined cereals (mean 17% of total fibre intake from non-wholegrain cereals) (OR = 2.83, 95% CI 1.089 – 7.333, *P* for trend = 0.021) with adjustments for age, sex, smoking, alcohol, and psychological morbidity (Model III). Conversely, participants in the highest quartile (Q_4_) of % fibre from nuts (mean 10% of total fibre intake from nut sources) were associated with 69% reduced odds of reporting *high* psoriasis severity (OR = 0.31, 95% CI 0.123 – 0.780, *P* for trend = 0.043). Both associations were no longer significant upon adjustment for BMI.

### Free sugars

Compared to those in the lowest quartile (Q_1_), participants in the highest quartile of intake (Q_4_) for % free sugars from beverages (mean 64% of total free sugar intake from beverage sources) were more likely to report a *high* psoriasis severity (OR = 3.85, 95% CI 1.507–9.831, *P* for trend = 0.040), when adjusted for age, sex, smoking, alcohol consumption, and psychological morbidity (Model III).

### Omega-6

As observed with % protein intakes, the directions of associations with psoriasis severity for % intakes of *n*-6 PUFAs differed according to animal and plant-based sources (refer to Supplementary Tables 9–10).

When adjusting for age, sex, smoking, alcohol consumption, and psychological morbidity (Model III), participants who obtained a greater proportion of *n*-6 from processed meat (Q_4_, mean 10% of total *n*-6 intake) were three-fold more likely to report *high* psoriasis severity (OR = 3.01, 95% CI 1.236–7.329, *P* for trend = 0.004) compared to those in the lowest quartile (Q_1_). On the other hand, participants obtaining greater proportions of *n*-6 PUFAs from nuts (Q_4_, mean 67% of *n*-6 intake) were less likely to report *high* psoriasis severity (OR = 0.27, CI 0.104–0.677, *P* for trend = 0.021).

The unadjusted ORs (95% CIs) of reporting a *high* psoriasis severity (T_3_ of sa-SPI score) and *increasing* psoriasis severity (T_2_ of sa-SPI score) across quartiles of % macronutrient intakes from food groups that did *not* exhibit statistically significant associations are reported in Supplementary Tables 11–14.

## Discussion

This study aimed to describe the macronutrient intakes of people living with psoriasis and explore associations with psoriasis severity. Overall, the intakes of the APPLE study population did not markedly differ from a nationally representative UK sample. Consumption of free sugars in this study population exceeded dietary guidelines [25] and was higher than that of the UK population (by approximately 2%). Intakes of dietary fibre aligned with the UK population intake (~ 20g/day), which is below the recommended intake (≥ 30g/day). Associations between proportions of macronutrients consumed from different food groups and psoriasis severity were dependent on the source, with plant-based sources of nutrients (protein, *n*-6 PUFAs, and fibre) observed to be generally associated with lower likelihoods of reporting higher psoriasis severity.

Higher proportions of free sugars from beverages (but not from breakfast cereals, desserts, or added sugars and condiments) was associated with a greater likelihood of reporting disease severity. This association disappeared after adjusting for BMI, suggesting that sugar-sweetened beverages could be a key target for dietary intervention in people who are overweight and living with psoriasis. Higher proportions of fibre from potato products and non-wholegrain cereals were associated with an increased likelihood of reporting higher psoriasis severity. This finding should be treated with caution, given the ‘potato product’ variable compounded intakes of jacket potatoes with crisps and chips, which differ in fibre content [23], whereby fibre from non-wholegrain foods is likely indicative of a poor diet, where a greater proportion of fibre is sourced from low-fibre foods. On the other hand, higher proportions of fibre from tree nut sources were associated with 69% reduced odds of reporting high psoriasis severity, another relationship appearing to be BMI-dependent.

In view of the case–control evidence presenting an elevated consumption of soft drinks and sugars [10, 31], and lower nut and total fibre intakes in psoriasis populations [11, 32], our findings reinforce the need for recommendations in this population group to address sugar excess and promote fibre intake. The consumption of dietary fibres can target appetite, which is suggested to be dysregulated in people with elevated BMIs [33], by promoting the secretion of satiety hormones [34]. Furthermore, the microbial fermentation of fibres sourced in nuts, fruits, and vegetables generates favourable immunomodulatory metabolites, e.g. short-chain fatty acids, polyamines, and tryptophan [35–37] whose role in psoriasis-related pathways has not been elucidated. Case–control evidence, however, has suggested a reduced abundance of *Bacteroidetes,* a fibre-degrading phylum [38], in people with psoriasis [39–42], where a diet low in fibre may hinder *Bacteroidetes* growth [43–45], with the host therefore failing to benefit from its metabolites [46].

Gut microbial metabolism of animal-sourced foods may also be a mediating factor accounting for the associations observed between greater proportions of protein and *n*-6 PUFAs derived from red and total meat and high psoriasis severity. Branched-chain amino acids (BCAAs) and Trimethylamine *N*-oxide (TMAO) are gut metabolites linked to systemic diseases such as T2D [47] and CVD [48] as well as inflammation [49], potentially explaining the opposing direction of associations between meat- or plant-derived protein, and *n*-6 PUFA intakes, with psoriasis severity. Males with psoriasis reportedly consume more red meat compared to healthy controls, according to results of an Italian case–control analysis [11], and red meat consumption has been linked to psoriasis severity in a mixed-sex Thai population (n = 100) [31]. Although both case–control analyses adopted the Psoriasis Area Severity Index as the disease severity measure, which, compared to the self-reported sa-SPI adopted in this study, is more objective as it is conducted by a clinician [50], the small sample sizes preclude the broader representation of these findings across psoriasis populations.

Studies have reported elevated concentrations of microbial metabolites such as serum TMAO [51–53] and serum BCAAs [54], as well as advanced glycation end products (AGEs) in the skin of people with severe psoriasis [55]. This evidence, however, is observational and lacks dietary assessment, making it challenging to establish causal links between higher consumption of animal-derived foods, greater TMAO, BCAA, and AGE concentrations, and greater psoriasis severity. Research has generally been gravitating towards the gut microbiome as a mediating factor for psoriasis onset and severity [56, 57]. Considering the lower microbial diversity and dysbiosis, characterised by higher *Firmicutes* and lower *Bacteroidetes* abundances, in people living with psoriasis, as systematically reviewed by Gao et al*.* [58], future psoriasis research must consider potential gut-skin axis interactions and their modulation by diet [59, 60].

### Strengths and limitations

To our knowledge, this is the first cross-sectional study profiling the macronutrient and food group intakes of a study population with psoriasis in the UK using a validated questionnaire measure for psoriasis severity. Limitations include incomplete survey responses, reducing the overall sample size available for analysis and the nature of participant convenience sampling, meaning that the findings may not be representative of the wider psoriasis population. Diet and psoriasis severity were self-reported, subject to recall, intake, and person-specific bias [61, 62]. Dietary intakes of the APPLE study population were based on FFQ data capturing semi-quantitative intakes across a multitude of food groups over a previous 12-month period. On the other hand, the NDNS intakes in Y9-11 are derived from 4-day food diaries, reflecting short-term day-to-day quantitative intakes of specific foods and meals, but potentially missing intakes of less frequently consumed foods [63]. Although the models accounted for BMI, adjustment for comorbidities was limited to psychological morbidity (self-reported depression and/or anxiety). Future cross-sectional studies should consider examining the relationship between diet and psoriasis severity in population subsets with cardiometabolic disease, as such comorbidities may influence psoriasis severity and dietary choices [64]. Additionally, insufficient data quality for exercise levels precluded adjustment for physical activity as a covariate. With a cross-sectional study design, cause-and-effect relationships cannot be established. Longitudinal studies may provide better insight into whether diet influences the disease course of psoriasis over time, while intervention trials incorporating dietary assessment and quantitative nutritional biomarker analyses are required to investigate causal relationships.

## Conclusion

These observations generate a plausible hypothesis that deriving a greater proportion of nutrients from plant sources may play a role in the management of psoriasis symptoms. The potential benefits of plant-based diets for people living with psoriasis remain an open question for future randomised controlled trials. Proportions of macronutrients from food sources may act as surrogate markers of other components in these foods that may be mechanistically implicated in the amelioration or worsening of psoriasis symptoms.

## Supplementary Information

Below is the link to the electronic supplementary material.

Supplementary Material 1

Supplementary Material 2

Supplementary Material 3

## Data Availability

The datasets generated and analysed during this study are available from the corresponding author on reasonable request, subject to any applicable ethical approvals and data sharing agreements.

## Appendix 1

### Survey design

The survey was designed on Qualtrics International Inc (https://www.qualtrics.com/uk/) and consisted of 14 sections. Before launching the study, the survey was piloted by the research team, medical professionals, and lay people with psoriasis (n = 8) to collect feedback on the questions, language, and survey logic. Revisions to the survey were implemented per the feedback to generate a 131-item survey.

### Survey development

Completeness checks were present at item level. Answers to previous questions could be reviewed by pressing the back button. Survey responses were auto-saved using cookies. If a survey was terminated, the survey could be resumed at the point of termination by clicking on the survey link, on the same browser and same device, within 7 days of starting the survey, provided cookie data was not deleted. After 7 days, survey responses were recorded on Qualtrics as incomplete. Upon survey submission, answers could not be amended. Participants could withdraw their data upon request.

### Survey responses

Participation in the study was voluntary, could be terminated at any time and required informed consent. To consent, volunteers indicated to have read the information sheet and agreed to each individual informed consent statement by clicking individual checkboxes. The survey logic would not grant access to the survey without consent. Participants self-reported their eligibility. As recompense for completing the APPLE study, participants were invited to attend a “Nutrition in Psoriasis” webinar.

### Data management

Only SZ had access to the identifiable information (name and email address) of the respondents. Survey responses were pseudonymised and assigned a unique identifier using a pseudonym code break spreadsheet. The pseudonym code break spreadsheet was password-protected and stored on a SharePoint drive, accessible only by SZ and the Principal Investigator (WLH).

### Data checks

To identify duplicate entries, participant IP addresses were scanned. Duplicate entries with the same IP address were eliminated before analysis. The initial, most complete response of a duplicate entry was retained for analysis. Incomplete survey responses (less than 100%) were excluded from this analysis.

### Survey metrics

Unique site visitors = counting the total number of unique IP addresses that accessed the survey.

View rate = dividing the number of respondents who clicked to have read the information sheet by the total number of unique site visitors.

Participation rate = dividing the number of respondents who provided informed consent by the number of respondents who clicked to have read the information sheet.

Completion rate = dividing the number of respondents who completed the survey by the number of respondents who provided informed consent.

## Appendix 2

### Food group composition according to Food Frequency Questionnaire items

**Table:**

Protein	
Total meat	Beef roast or steak or mince, or stew or casserole, beef burgers, pork roast or chops or stew, lamb roast or chops or stew, chicken or other poultry, bacon or gammon, ham or cured meats or chorizo, corned beef or spam or luncheon meats, sausages, liver or liver pâté or liver sausage
Red meat	Beef roast or steak or mince, or stew or casserole, pork roast or chops or stew, lamb roast or chops or stew, liver or liver pâté or liver sausage
Processed meat	Beef burgers, bacon or gammon, ham or cured meats or chorizo, corned beef or spam or luncheon meats, sausages
Poultry	Chicken or other poultry
Eggs	Eggs as boiled, fried, or scrambled
Fish	Fried fish in batter, as fish and chips, fish fingers, fish cakes or breaded fish, other white fish, fresh or frozen, e.g. cod, plaice, sole, haddock or halibut, oily fish, fresh or canned, e.g. tuna, mackerel, kippers, salmon, sardines or herring, shellfish, e.g. crab, prawns or mussels, fish roe, taramasalata
High-fat dairy	Single or sour cream, double or clotted cream, full fat or Greek yoghurt, cheese, e.g. cheddar, brie, or edam
Low-fat dairy	Low fat yoghurt, fromage frais, low fat cheese e.g. reduced fat cheddar, cottage cheese or low-fat soft cheese
Plant-based sources	Peas, green beans, broad beans or runner beans, baked beans, bean sprouts, pulses e.g. lentils, beans, peas, meat substitutes e.g. tofu, soya meat, textured vegetable protein or vegeburger
Tree nuts	Salted nuts, e.g. peanuts, cashews, unsalted nuts, e.g. Brazil, walnuts, seeds e.g. Sunflower, pumpkin, peanut butter

**Table:**

Fibre	
Fruit	Apples, pears, oranges or satsumas or mandarins, grapefruit, bananas, grapes, melon, peaches or plums or apricots, strawberries or raspberries or kiwi fruit, tinned fruit, dry fruit, avocado
Vegetables	Carrots, spinach, broccoli or spring greens or kale, brussels sprouts, cabbage, marrow or courgettes, cauliflower, parsnips or turnips or swedes, leeks, onions, garlic, mushrooms, sweet peppers, green salad or lettuce or cucumber or celery, watercress, tomatoes, sweetcorn, beetroot
Potato products	Boiled, mashed, instant or jacket potato, chips or roast potatoes, potato salad, crisps or other packet snacks
Legumes	Peas, green beans or broad beans or runner beans, baked beans, beansprouts, dried lentils or beans or peas
Wholegrain cereals	Brown bread rolls, wholemeal and granary bread and rolls, porridge, muesli, high-fibre cereal, brown rice, wholemeal pasta
Non-wholegrain cereals	White bread and rolls, cream crackers or savoury biscuits, crispbread, naan or poppadoms or flour tortillas, breakfast cereal, sugar-topped breakfast cereal, white rice, white or green pasta, reduced fat biscuits, cereal bars
Tree nuts	Salted nuts, unsalted nuts, seeds, peanut butter

**Table:**

Free sugars	
Desserts & puddings	Sweet biscuits (chocolate & plain), reduced fat biscuits, cereal bars, cakes (home-baked and ready-made), buns and pastries (home-baked and ready-made) fruits pies or tarts or crumbles (home-baked and ready-made) sponge puddings (home-baked and ready-made), milk puddings, ice cream or choc ices, dairy desserts, white or milk chocolates, dark chocolates, chocolate snacks, sweets, toffees or mints
Beverages	Cocoa hot chocolate, low fat hot chocolate, horlicks or ovaltine, low calorie or diet fizzy soft drinks, fizzy soft drinks, pure fruit juice, fruit squash or cordial, smoothies
Breakfast cereal	Breakfast cereal, sugar topped cereals, muesli, high-fibre cereals
Added sugars	Sugar added to tea or coffee or cereal, tomato ketchup, pickles or chutney, jam or marmalade or honey

**Table:**

Omega-6	
Tree nuts	Salted nuts, e.g. peanuts, cashews, unsalted nuts, e.g. Brazil, walnuts, seeds e.g. Sunflower, pumpkin, peanut butter
Spreads	Butter, reduced fat butter, block margarine, polyunsaturated margarine, olive oil spread, other soft margarine or dairy spreads, low fat spread, very low-fat spread, cholesterol lowering fat spreads
Red meat	Beef roast or steak or mince, or stew or casserole, pork roast or chops or stew, lamb roast or chops or stew, liver or liver pâté or liver sausage
Processed meat	Beef burgers, bacon or gammon, ham or cured meats or chorizo, corned beef or spam or luncheon meats, sausages
Poultry	Chicken or other poultry
Eggs	Eggs as boiled, fried, or scrambled
