# Supplementary material for: Macronutrient intakes and associations with psoriasis severity: a cross-sectional analysis of the asking people with psoriasis about lifestyle and eating (APPLE) study
Source: Eur J Nutr. 2026 Feb 19;65(2):64. doi: 10.1007/s00394-026-03914-y (PMC12920720; doi:10.1007/s00394-026-03914-y)
Supplement: Supplementary file 1 — Supplementary Material 1 [file 394_2026_3914_MOESM1_ESM.docx]

**Article title:** Macronutrient intakes and associations with psoriasis severity: a cross-sectional analysis of the Asking People with Psoriasis about Lifestyle and Eating (APPLE) study.

**Journal name:** European Journal of Nutrition.

**Authors:** Sylvia Zanesco, Thiviyani Maruthappu, Christopher E.M. Griffiths, Ruotong Zhang, Kathryn V. Dalrymple, Rachel Gibson, and Wendy L. Hall.

# **Corresponding author:** Sylvia Zanesco, Department of Nutritional Sciences, King's College London, UK, (sylvia.1.zanesco@kcl.ac.uk).

# **Supplementary Table 1.** Strengthening the Reporting of Observational Studies in Epidemiology – Nutrition checklist.

| **Reporting Item** | | **Page number** |
| --- | --- | --- |
| **Title and abstract** |  |  |
| Title | Indicate the study’s design with a commonly used term in the title or the abstract | 1 |
| None | State the dietary/nutritional assessment method(s) used in the title or in the abstract. | 1 |
| Abstract | Provide in the abstract an informative and balanced summary of what was done and what was found | 1-2 |
| **Introduction** |  |  |
| Background / rationale | Explain the scientific background and rationale for the investigation being reported | 3-4 |
| Objectives | State specific objectives, including any prespecified hypotheses | 4 |
| **Methods** |  |  |
| Study design | Present key elements of study design early in the paper | 4 |
| Setting | Describe the setting, locations, and relevant dates, including periods of recruitment, exposure, follow-up, and data collection | 4 |
| None | Describe any characteristics of the study settings that might affect the dietary intake or nutritional status of the participants, if applicable. | N/A |
| Eligibility | Cross-sectional study: Give the eligibility criteria, and the sources and methods of selection of participants. | 4 |
| None | Report any particular dietary, physiologic, or nutritional characteristics that were considered when selecting the target population. | N/A |
| Variables | Clearly define all outcomes, exposures, predictors, potential confounders, and effect modifiers. | 5-8 |
| None | Clearly define foods, food groups, nutrients, or other food components (e.g., preparation method, taxonomical descriptors, classification, chemical form). |  |
| None | When calculating dietary patterns, describe the methods to obtain them and their nutritional properties. | N/A |
| Data sources and measurement | For each variable of interest give sources of data and details of methods of assessment (measurement). | 5 |
|  | Describe the dietary assessment method(s) (e.g., portion size estimation, number of days and items recorded, how it was developed and administered, and how quality was ensured); report if and how supplement intake was assessed. | 5 |
|  | Describe and justify food-composition data used; explain the procedure to match food composition with consumption data; describe the use of conversion factors, if applicable | 5 |
| None | Describe the nutrient requirements, recommendations, or dietary guidelines and the evaluation approach used to compare intake with the dietary reference values, if applicable | 6 |
| None | When using nutritional biomarkers, additionally use the STROBE-ME; report the type of biomarkers used and usefulness as dietary exposure markers | N/A |
| None | Describe the assessment of nondietary data (e.g., nutritional status and influencing factors) and timing of the assessment of these variables in relation to dietary assessment | N/A |
| None | Report on the validity of the dietary or nutritional assessment methods and any internal or external validation used in the study. | N/A |
| Bias | Describe any efforts to address potential sources of bias | 5,7 |
| None | Report how bias in dietary or nutritional assessment was addressed | 5,7 |
| Study size | Explain how the study size was arrived at | N/A |
| Quantitative variables | Explain how quantitative variables were handled in the analyses. If applicable, describe which groupings were chosen, and why | 6-7 |
| None | Explain categorization of dietary/nutritional data (e.g., use of N-tiles and handling of non-consumers) and the choice of reference category, if applicable. | 6 |
| Statistical methods | Describe all statistical methods, including those used to control for confounding | 7-8 |
| Subgroups and interactions | Describe any methods used to examine subgroups and interactions | N/A |
| Missing data | Explain how missing data were addressed | **Appendix 1** |
| Loss to follow up | Cohort study: if applicable, explain how loss to follow-up was addressed. Case-control study: if applicable, explain how matching of cases and controls was addressed. Cross-sectional study: if applicable, describe analytical methods taking account of sampling strategy. | N/A |
| Sensitivity analysis | Describe any sensitivity analyses | N/A |
|  | Describe any statistical method used to combine dietary or nutritional data, if applicable. | N/A |
|  | Describe and justify the method for energy adjustments, intake modeling, and use of weighting factors, if applicable | 6-7 |
| None | Report any adjustments for measurement error (i.e., from a validity or calibration study). | N/A |
| **Results** |  |  |
| Participants | Report numbers of individuals at each stage of study—eg numbers potentially eligible, examined for eligibility, confirmed eligible, included in the study, completing follow-up, and analysed. Give information separately for exposed and unexposed groups if applicable. | 8-9 |
| Non-participation | Give reasons for non-participation at each stage |  |
| Participant journey | Consider the use of a flow diagram |  |
| None | Report the number of individuals excluded on the basis of missing, incomplete, or implausible dietary and nutritional data. | 8-9 |
| Descriptive data | Give characteristics of study participants (eg demographic, clinical, social) and information on exposures and potential confounders. Give information separately for exposed and unexposed groups if applicable. | 9-11 |
| Missing data | Indicate number of participants with missing data for each variable of interest | **Table 1 and Supplementary Table 3** |
| Follow-up time | Cohort study: Summarise follow-up time (eg, average and total amount) | N/A |
| None | Give the distribution of participant characteristics across the exposure variables, if applicable; specify if food consumption for the total population or consumers only was used to obtain results | specified in the tables |
| Outcome data | Cross-sectional study: report numbers of outcome events or summary measures. | N/A |
| Main results | Give unadjusted estimates and, if applicable, confounder-adjusted estimates and their precision (eg, 95% confidence interval). Make clear which confounders were adjusted for and why they were included | **Table 4** and **Supplementary Tables 7-4** |
| Category boundaries | Report category boundaries when continuous variables were categorized | specified in the footnotes of tables |
| Relative and absolute risks | If relevant, consider translating estimates of relative risk into absolute risk for a meaningful time period | N/A |
| None | Specify if nutrient intakes are reported with or without the inclusion of dietary supplement intake, if applicable. | N/A |
| Other analyses | Report other analyses done—eg analyses of subgroups and interactions, and sensitivity analyses | N/A |
| None | Report any sensitivity analysis (e.g., exclusion of misreporters or outliers) and data imputation, if applicable | N/A |
| **Discussion** |  |  |
| Key results | Summarise key results with reference to study objectives | 22 |
| Limitations | Discuss limitations of the study, taking into account sources of potential bias or imprecision. Discuss both direction and magnitude of any potential bias. | 23-24 |
|  | Describe the main limitations of the data sources and assessment methods used and implications for the interpretation of the findings |  |
| Interpretation | Give a cautious overall interpretation considering objectives, limitations, multiplicity of analyses, results from similar studies, and other relevant evidence. | 22-23 |
|  | Report the nutritional relevance of the findings, given the complexity of diet or nutrition as an exposure. |  |
| Generalisability | Discuss the generalisability (external validity) of the study results | 23 |
| **Other Information** |  |  |
| Funding | Give the source of funding and the role of the funders for the present study and, if applicable, for the original study on which the present article is based | 3 |
| Ethics | Describe the procedure for consent and study approval from ethics committee(s). | **Appendix 1** and page 4 |
| Data statement | Provide data collection tools and data as online material or explain how they can be accessed. | As per journal requirements. |

This checklist was adopted from: Lachat C, Hawwash D, Ocké MC, Berg C, Forsum E, Hörnell A, et al. Strengthening the Reporting of Observational Studies in Epidemiology – nutritional epidemiology (STROBE-nut): An extension of the STROBE statement. Nutr Bull. 2016 Sep 1;41(3):240–51.
